# Near Infrared Photoimmunotherapy Targeting EGFR Positive Triple Negative Breast Cancer: Optimizing the Conjugate-Light Regimen

**DOI:** 10.1371/journal.pone.0136829

**Published:** 2015-08-27

**Authors:** Tadanobu Nagaya, Kazuhide Sato, Toshiko Harada, Yuko Nakamura, Peter L. Choyke, Hisataka Kobayashi

**Affiliations:** Molecular Imaging Program, Center for Cancer Research, National Cancer Institute, Bethesda, Maryland, United States of America; AntiCancer Inc., UNITED STATES

## Abstract

**Aim:**

Triple-negative breast cancer (TNBC) is considered one of the most aggressive subtypes of breast cancer. Near infrared photoimmunotherapy (NIR-PIT) is a cancer treatment that employs an antibody-photosensitizer conjugate (APC) followed by exposure of NIR light for activating selective cytotoxicity on targeted cancer cells and may have application to TNBC. In order to minimize the dose of APC while maximizing the therapeutic effects, dosing of the APC and NIR light need to be optimized. In this study, we investigate *in vitro* and *in vivo* efficacy of cetuximab (cet)-IR700 NIR-PIT on two breast cancer models MDAMB231 (TNBC, EGFR moderate) and MDAMB468 (TNBC, EGFR high) cell lines, and demonstrate a method to optimize the dosing APC and NIR light.

**Method:**

After validating *in vitro* cell-specific cytotoxicity, NIR-PIT therapeutic effects were investigated in mouse models using cell lines derived from TNBC tumors. Tumor-bearing mice were separated into 4 groups for the following treatments: (1) no treatment (control); (2) 300 μg of cet-IR700 i.v., (APC i.v. only); (3) NIR light exposure only, NIR light was administered at 50 J/cm^2^ on day 1 and 100 J/cm^2^ on day 2 (NIR light only); (4) 300 μg of cet-IR700 i.v., NIR light was administered at 50 J/cm^2^ on day 1 after injection and 100 J/cm^2^ of light on day 2 after injection (one shot NIR-PIT). To compare different treatment regimens with a fixed dose of APC, we added the following treatments (5) 100 μg of cet-IR700 i.v., NIR light administered at 50 J/cm^2^ on day 1 and 50 μg of cet-IR700 i.v. immediately after NIR-PIT, then NIR light was administered at 100 J/cm^2^ on day 2, which were performed two times every week (“two split” NIR-PIT) and (6) 100 μg of cet-IR700 i.v., NIR light was administered at 50 J/cm^2^ on day 1 and 100 J/cm^2^ on day 2, which were performed three times per week (“three split” NIR-PIT).

**Result:**

Both specific binding and NIR-PIT effects were greater with MDAMB468 than MDAMB231 cells *in vitro*. Tumor accumulation of cet-IR700 in MDAMB468 tumors was significantly higher (*p* < 0.05) than in MDAMB231 tumors *in vivo*. Tumor growth and survival of MDAMB231 tumor bearing mice was significantly lower in the NIR-PIT treatment group (*p* < 0.05). In MDAMB468 bearing mice, tumor growth and survival was significantly improved in the NIR-PIT treatment groups in all treatment regimens (one shot NIR-PIT; *p* < 0.05, “two split” NIR-PIT; *p* < 0.01, “three split” NIR-PIT; *p* < 0.001) compared with control groups.

**Conclusion:**

NIR-PIT for TNBC was effective regardless of expression of EGFR, however, greater cell killing was shown with higher EGFR expression tumor *in vitro*. In all treatment regimens, NIR-PIT suppressed tumor growth, resulting in significantly prolonged survival that further improved by splitting the APC dose and using repeated light exposures.

## Introduction

Breast cancer is generally classified based on the receptors overexpressed on the cancer cell membrane, such as estrogen receptor (ER), progesterone receptor (PR) and human epidermal growth factor receptor 2 (HER2). According to the expression status of these receptors, breast cancers are classified as luminal A (ER+ or PR+/HER2-), luminal B (ER+ or PR+/HER2+), HER2 (ER-/PR-/HER2+) or triple-negative breast cancer (TNBC, ER-/PR-/HER2-) [1,2]. TNBC is further subclassified as basal-like breast cancer (BLBC, ER-/PR-/HER2-/CK5/6+or epidermal growth factor receptor [EGFR] +) and quintuple-negative breast cancer (QNBC, ER-/PR-/HER2-/CK5/6-/EGFR-) and accounts for 15–25% of all breast cancer cases [3]. TNBC is associated with shorter relapse-free intervals and lower overall survival than the other subtypes [4,5]. Moreover, no targeted therapy is currently available for TNBC, in contrast to ER-positive or HER2-positive breast tumors. Thus there is a need to define new therapy which will be active in this aggressive breast cancer subtype.

EGFR is part of the human epidermal growth factor receptor family and can form heterodimers with HER2 and other HER family members. Activation of these receptors results in accelerated cell growth and carcinogenesis [6]. EGFR has been shown to be overexpressed in a substantial percentage of triple negative breast cancers [7] and has been viewed as a promising therapeutic target. Cetuximab, chimeric (mouse/human) monoclonal antibody, has been approved by the US FDA and have been widely used for EGFR-expressing tumors. [8] Unfortunately, the therapeutic efficacy of EGFR-targeting agents has been limited in TNBC [9,10].

Near infrared photoimmunotherapy (NIR-PIT) is a new cancer treatment based on an antibody-photosensitizer conjugate (APC). The photosensitizer, IRDye700DX, which is a water-soluble silica-phthalocyanine dye is conjugated to an antibody to form an APC. APC binds to its cell surface target and induces cytotoxicity after exposure of NIR light at a wavelength 690 nm. *In vitro* studies have shown NIR-PIT to be highly cell-specific, therefore, non-target expressing cells immediately adjacent to targeted cells show no toxic effect [11]. Cell membrane rupture is induced shortly after the exposure of NIR-light to target cells indicating a rapid necrotic cell death. NIR-PIT is potentially effective in a broad range of cancers given the large number of cell surface receptors, their cognate antibodies and the facile chemistry of conjugating them to a photosensitizer [11–16].

One issue regards the correct dosing of the APC and NIR light, both of which are variables that must be fixed for clinical trials. In this study, we investigate *in vitro* and *in vivo* cell killing efficacy of NIR-PIT using TNBC cell lines, MDAMB231 (EGFR moderate) and MDAMB468 (EGFR high) [17]. Additionally, we investigate an optimal dosing regimen of a fixed amount of APC with various exposure of NIR light that is most effective for MDAMB468 tumors.

## Material and Methods

### Reagents

Cetuximab-IR700 (cet-IR700) was obtained from the Imaging Probe Development Center (Rockville, MD, USA). IRDye 800CW NHS ester (IR800; C_50_H_54_N_3_Na_3_O_17_S_4_, molecular weight of 1166.2030) was purchased from LI-COR Biosciences (Lincoln, NE, USA). Purified mouse IgG was purchased from SIGMA (Saint Louis, MO, USA). All other chemicals were of reagent grade.

### Synthesis of IR800-conjugated mouse IgG (mouse IgG-IR800)

Conjugation of dyes with mouse IgG was performed according to the procedure reported previously [11]. IgG (0.75 mg) was incubated with IR800 (35.8 μg, 30.8 nmol, 5 mmol/L in DMSO) in 0.1 M Na2HPO4 (pH 8.6) at room temperature for 1 h. The mixture was purified with a Sephadex G25 column (PD-10; GE Healthcare, Piscataway, NJ, USA). The protein concentration was determined with Coomassie Plus protein assay kit (Thermo Fisher Scientific Inc, Rockford, IL, USA) by measuring the absorption at 595 nm with spectroscopy (8453 Value System; Agilent Technologies, Santa Clara, CA, USA). The concentration of IR800 was measured by absorption at 774 nm with spectroscopy to confirm the number of fluorophore molecules conjugated to mAbs. The number of IR800 per antibody was adjusted to approximately two.

### Cell culture

MDAMB231 and MDAMB468 cells were obtained from National Cancer Institute (NCI)-Frederick Cancer Division of Cancer Treatment and Diagnosis (DCTD) Tumor/Cell Line Repository (Frederick, MD, USA). Cells were grown in PRMI 1640 (Life Technologies, Gaithersburg, MD, USA) supplemented with 10% fetal bovine serum and 1% penicillin/streptomycin (Life Technologies) in tissue culture flasks in a humidified incubator at 37°C at an atmosphere of 95% air and 5% carbon dioxide.

### Fluorescence Microscopy

To detect the antigen specific localization and effect of NIR-PIT, fluorescence microscopy was performed (BX61; Olympus America, Inc., Melville, NY, USA). Ten thousand cells were seeded on cover-glass-bottomed dishes and incubated for 48 h. Cet-IR700 was then added to the culture medium at 10 μg/ml and incubated for 6 h at 37°C. After incubation the cells were washed with phosphate buffered saline (PBS). The filter set to detect IR700 consisted of a 590–650 nm excitation filter, a 665–740 nm band pass emission filter.

### Flow cytometry

Fluorescence from cells after incubation with cet-IR700 was measured using a flow cytometer (FACS Calibur, BD BioSciences, San Jose, CA, USA) and CellQuest software (BD BioSciences). MDAMB231 and MDAMB468 cells (1 × 10^5^) were seeded into 24 well plates and incubated for 24 h. Medium was replaced with fresh culture medium containing 10 μg/ml of cet-IR700 and incubated for 6 h at 37°C. To validate the specific binding of the conjugated antibody, excess antibody (100 μg) was used to block the dye-antibody conjugates.

### *In vitro* NIR-PIT

Two hundred thousand cells were seeded into 12 well plates and incubated for 24 h. Medium was replaced with fresh culture medium containing 10 μg of cet-IR700 and incubated for 6 h at 37°C. After washing with PBS, PBS was added. Then, cells were irradiated with a red light-emitting diode (LED), which emits light at 670-710nm wavelength (L690-66-60; Marubeni America Co., Santa Clara, CA, USA), with a power density of 50 mW/cm^2^ as measured with an optical power meter (PM 100, Thorlabs, Newton, NJ, USA).

### Cytotoxicity/phototoxicity assay

The cytotoxic effects of NIR-PIT with cet-IR700 were determined by flow cytometric Propidium Iodide (PI) (Life Technologies) staining, which can detect compromised cell membranes. Cells were scratched 1 h after treatment. PI was added in the cell suspension (final 2 μg/ml) and incubated at room temperature for 30 min, followed by flow cytometry. Each value represents mean ± s.e.m. of five experiments.

### Animal and tumor models

All in vivo procedures were conducted in compliance with the Guide for the Care and Use of Laboratory Animal Resources (1996), US National Research Council, and approved by the Animal Care and Use Committee in the National Cancer Institute. Six- to eight-week-old female homozygote athymic nude mice were purchased from Charles River (NCI-Frederick, Frederick, MD). During the procedure, mice were anesthetized with isoflurane. In order to determine tumor volume, the greatest longitudinal diameter (length) and the greatest transverse diameter (width) were measured with an external caliper. Tumor volumes were based on caliper measurements and were calculated using the following formula; tumor volume = length × width^2^ × 0.5 [18]. Body weight (BW) was also measured. Mice were monitored daily in health condition and tumor volumes were measured three times a week until the tumor volume reached 4000 mm^2^, whereupon the mice were euthanized with inhalation of carbon dioxide gas. We did not observe any findings of distresses including weight loss, appetite loss, and ulceration on tumors, in any groups of mice in this study.

### *In vivo* fluorescence imaging studies

MDAMB231 cells (4.5 × 10^6^) were injected subcutaneously in the left dorsum and MDAMB468 cells (6 × 10^6^) were injected subcutaneously in the right dorsum of the same mice. Sixteen days after MDAMB231 and MDAMB468 cell injection, tumors reached volumes of approximately 50 mm^3^ and those animals in which both tumors were approximately the same size were selected for the study. *In vivo* fluorescence images of IR700 and IR800 were obtained with a Pearl Imager (LI-COR Biosciences, Lincoln, Nebraska, USA) using the 700 and 800 nm fluorescence channel after intravenous injection of 100 μg of cet-IR700 and 20 μg of mouse IgG-IR800 conjugate. For analyzing fluorescence intensities, tumors of the same size were compared and regions of interest (ROI) were placed over the entire tumor and background. Average fluorescence intensity of each ROI was calculated. Tumor background ratios (TBR) (Fluorescence intensities of tumor/fluorescence intensities of background) were calculated. To compare the fluorescence between MDAMB231 and MDAMB468, TBR700/TBR800 were also calculated.

### *In vivo* NIR-PIT

#### One shot NIR-PIT regimen

MDAMB231 cells (6 × 10^6^) and MDAMB468 cells (6 × 10^6^) were injected subcutaneously in the right dorsum of the mice. Sixteen days later tumor volumes of approximately 50 mm^3^ were selected for the study. Tumor bearing mice were randomized into 4 groups of at least 10 animals per group for the following treatments: (1) no treatment (control); (2) 300 μg of cet-IR700 i.v., no NIR light exposure (APC i.v. only); (3) NIR light exposure only, NIR light was administered at 50 J/cm^2^ on day 1 and 100 J/cm2 on day 2 (NIR light only); (4) 300 μg of cet-IR700 i.v., NIR light was administered at 50 J/cm^2^ on day 1 after injection and 100 J/cm^2^ on day 2 after injection (PIT).

#### “Two split” NIR-PIT regimen

MDAMB468 cells (6 × 10^6^) were injected subcutaneously in the right dorsum of the mice. Sixteen days later, tumor volumes of approximately 50 mm^3^ were selected for the study. Tumor-bearing mice were randomized into 3 groups of at least 10 animals per group for the following treatments: (1) no treatment (control); (2) 100 μg of cet-IR700 i.v. followed by a weekly dose of 50 μg of cet-IR700, no NIR light exposure (APC i.v. only); (3) 100 μg of cet-IR700 i.v. and 50 μg of cet-IR700 i.v. immediately after NIR on day 1, NIR light was administered at 50 J/cm^2^ on day 1 and 100 J/cm^2^ on day 2. These therapies were performed every week for up to 2 weeks (“two split” NIR-PIT).

#### “Three split” NIR-PIT regimen

MDAMB468 cells (6 × 10^6^) were injected subcutaneously in the right dorsum of the mice. Sixteen days later, tumor volumes of approximately 50 mm^3^ were selected for the study. Tumor bearing mice were randomized into 4 groups of at least 10 animals per group for the following treatments: (1) no treatment (control); (2) 100 μg of cet-IR700 i.v. every week, no NIR light exposure (APC i.v. only); (3) NIR light exposure only, NIR light was administered at 50 J/cm^2^ on day 1 and 100 J/cm^2^ on day 2 (NIR light only); (4) 100 μg of cet-IR700 i.v. every week, NIR light was administered at 50 J/cm^2^ on day 1 after injection and 100 J/cm^2^ on day 2 after injection. These therapies were performed every week for up to 3 weeks (“three split” NIR-PIT).

Fluorescence images, as well as white light images, were obtained using a Pearl Imager with a 700 nm fluorescence channel. Tumor volumes and BW were measured two times a week until the tumor diameter reached 2 cm, whereupon the mice were euthanized with carbon dioxide.

### Statistical analysis

Data are expressed as means ± s.e.m. from a minimum of five experiments, unless otherwise indicated. Statistical analyses were carried out using a statistics program (GraphPad Prism; GraphPad Software, La Jolla, CA, USA). For multiple comparisons, a one-way analysis of variance (ANOVA) followed by the Bonferroni’s multiple comparisons test was used. The cumulative probability of survival based on volume (4000 mm^3^) were estimated in each group with a Kaplan-Meier survival curve analysis, and the results were compared with use of the log-rank test. To compare with intensity, Mann-Whitney-U test was used. Student’s t test was used to compare the treatment effects with that control. *P*-value of <0.05 was considered statistically significant.

## Result

### *In vitro* characterization of MDAMB231 and MDAMB468

**Fig 1:**
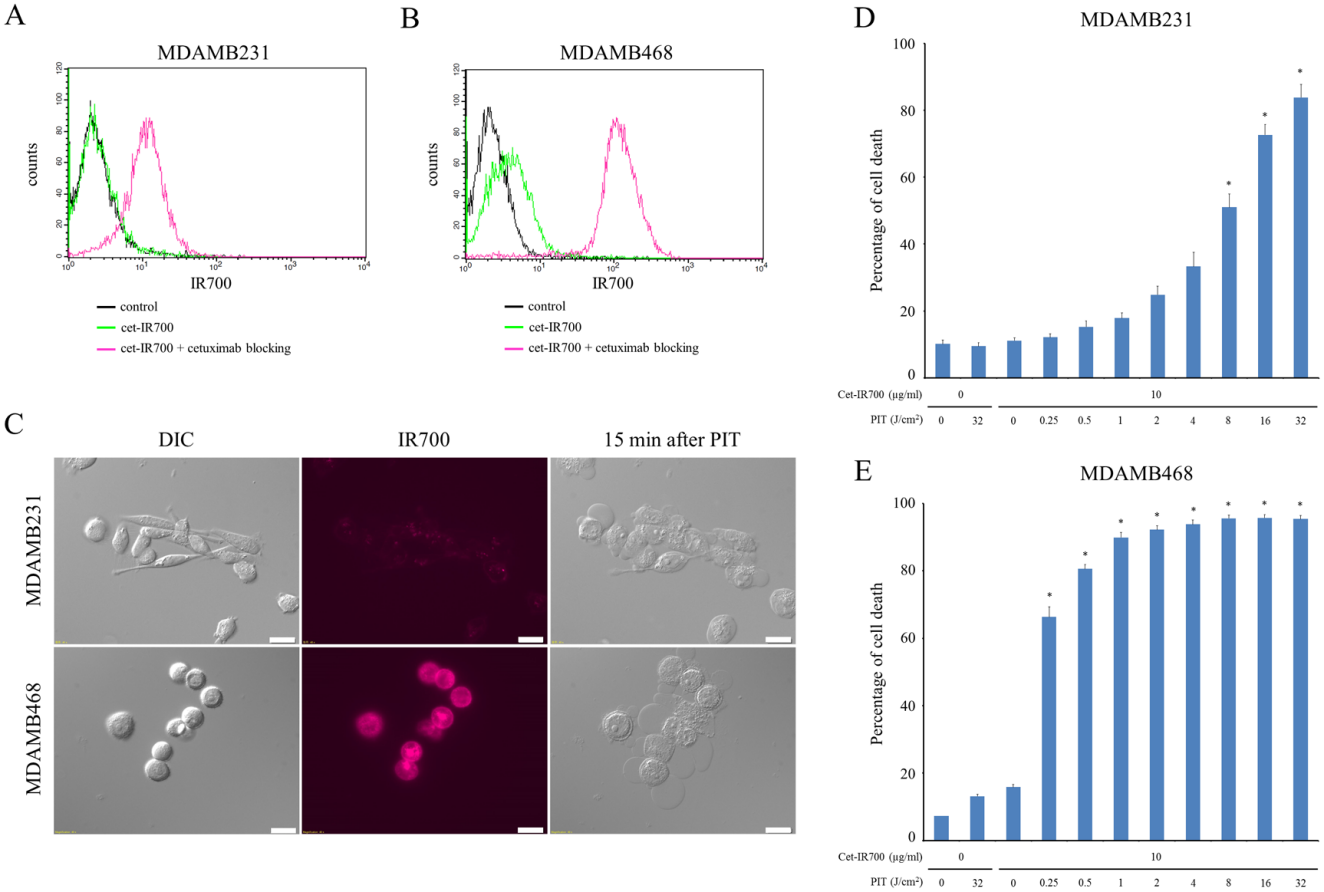
Confirmation of EGFR expression as a target for PIT in MDAMB231 and MDAMB468 cells, and evaluation of *in vitro* PIT. (A) Expression of EGFR in MDAMB231 cells was examined with FACS. (B) Expression of EGFR in MDAMB468 cells was examined with FACS. (C) MDAMB231 and MDAMB468 cells were incubated with cet-IR700 for 6 h and observed by microscopy. Fluorescence intensities of MDAMB468 were higher than MDAMB231. Necrotic cell death was observed upon excitation with 2 J/cm^2^ of NIR light (after 15min). Bar = 20 μm. (D) Membrane damage of MDAMB231 cells induced by PIT was measured with PI staining (dead cell count), which increased in a light dose-dependent manner (n = 5, *p < 0.001, vs. untreated control, by Student’s t test). (E) PI staining showed membrane damage of MDAMB468 cells (n = 5, *p < 0.001, vs. untreated control, by Student’s t test).

IR700 fluorescence signals analyzed by the flow cytometry showed that cet-IR700 bound to both MDAMB231 and MDAMB468 cells. After incubation with cet-IR700 both cells showed fluorescence signals in microscopic images (Fig 1A and 1B). Fluorescence signal intensities of MDAMB468 were higher than MDAMB231 on both methods above. These signals were completely blocked by adding excess cetuximab that indicated that the APC specifically bound to EGFR.

### *In vitro* NIR-PIT

Time lapse fluorescence microscopy imaging was performed 6 h after incubation with cet-IR700 to compare in vitro APC binding and cell killing efficacy of NIR-PIT on MDAMB231 and MDAMB468. Fluorescence intensities of MDAMB468 were higher than MDAMB231 (Fig 1C). Immediately after exposure, NIR light induced cellular swelling, bleb formation, and rupture of vesicles representing necrotic cell death in MDAMB468 cells. Although the necrotic cell death was also observed in MDAMB231 cells, this occurred more slowly and the number of necrotic cell deaths was fewer than with MDAMB468 during visual observation (S1 and S2 Videos). However, most of these morphologic changes were observed on both cell types within 15 min of light exposure (Fig 1C), indicating rapid induction of necrotic cell death.

In order to examine the effect of *in vitro* NIR-PIT quantitatively, we performed a cytotoxicity assay with PI staining (Fig 1D and 1E). Based on incorporation of PI, percentage of cell death increased in a light dose dependent manner. There was no significant cytotoxicity associated with NIR light alone in the absence of agent and with agent alone without NIR light. Percentage of cell death of MDAMB468 cells was higher than MDAMB231 at the same light doses. Although over 90% of MDAMB468 cells died when exposed to 1 J of PIT, percentage of cell death of MDAMB231 is about 85% after 32 J of PIT.

### *In vivo* fluorescence imaging studies

**Fig 2:**
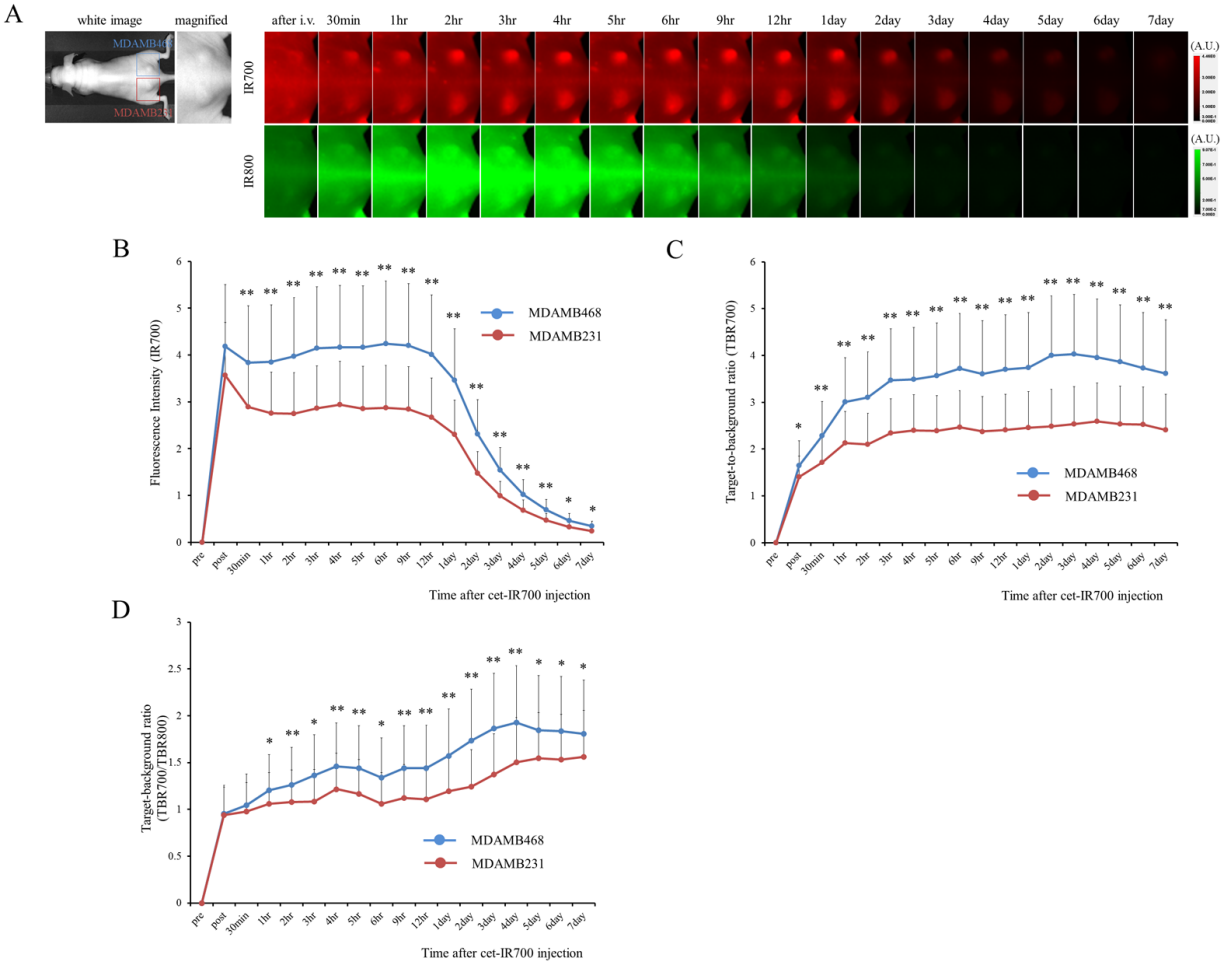
*In vivo* fluorescence imaging of MDAMB231 and MDAMB468 tumors. (A) *In vivo* cet-IR700 and mouse IgG-IR800 fluorescence real-time imaging of bilateral (right dorsum; MDAMB468, left dorsum; MDAMB231) flank tumors in mice. The fluorescence intensity of cet-IR700 and mouse IgG-IR800 in both MDAMB231 and MDAMB468 tumor decreased gradually over days. The fluorescence intensity of IR700 was higher in MDAMB468 tumor compared with MDAMB231 tumor, while the fluorescence intensity of IR800 was almost same in both tumors. (B) Quantitative analysis of IR700 intensities in both tumors. The fluorescence intensities were significantly higher in MDAMB468 tumors compared with MDAMB231 tumors (n = 10, *p < 0.05, **p < 0.01, by Mann-Whitney-U test). (C) Quantitative analysis of TBR in both tumors demonstrated differences between MDAMB468 tumors and MDAMB231 tumors (n = 10, *p < 0.05, **p < 0.01, by Mann-Whitney-U test). (D) Quantitative analysis of TBR700/TBR800 in both tumors. Intensities were significantly higher in MDAMB468 tumors compared with MDAMB231 tumors (n = 10, *p < 0.05, **p < 0.01, by Mann-Whitney-U test).

To quantitatively evaluate fluorescence intensities in tumor bearing mice fluorescence intensities and TBR were assessed (n = 10 mice) (Fig 2). The fluorescence intensity of cet-IR700 in both MDAMB231 and MDAMB468 tumor decreased gradually over days (Fig 2A and 2B), while TBR increased (Fig 2C). The fluorescence intensity, TBR and TBR700/TBR800 were significantly higher in MDAMB468 tumors compared to MDAMB231 tumors. These results suggest that tumor cell binding of cet-IR700 in MDAMB468 tumors was significantly higher (*p* < 0.05) than in MDAMB231 tumors *in vivo*.

### *In vivo* NIR-PIT: one shot regimen for two tumor models

**Fig 3:**
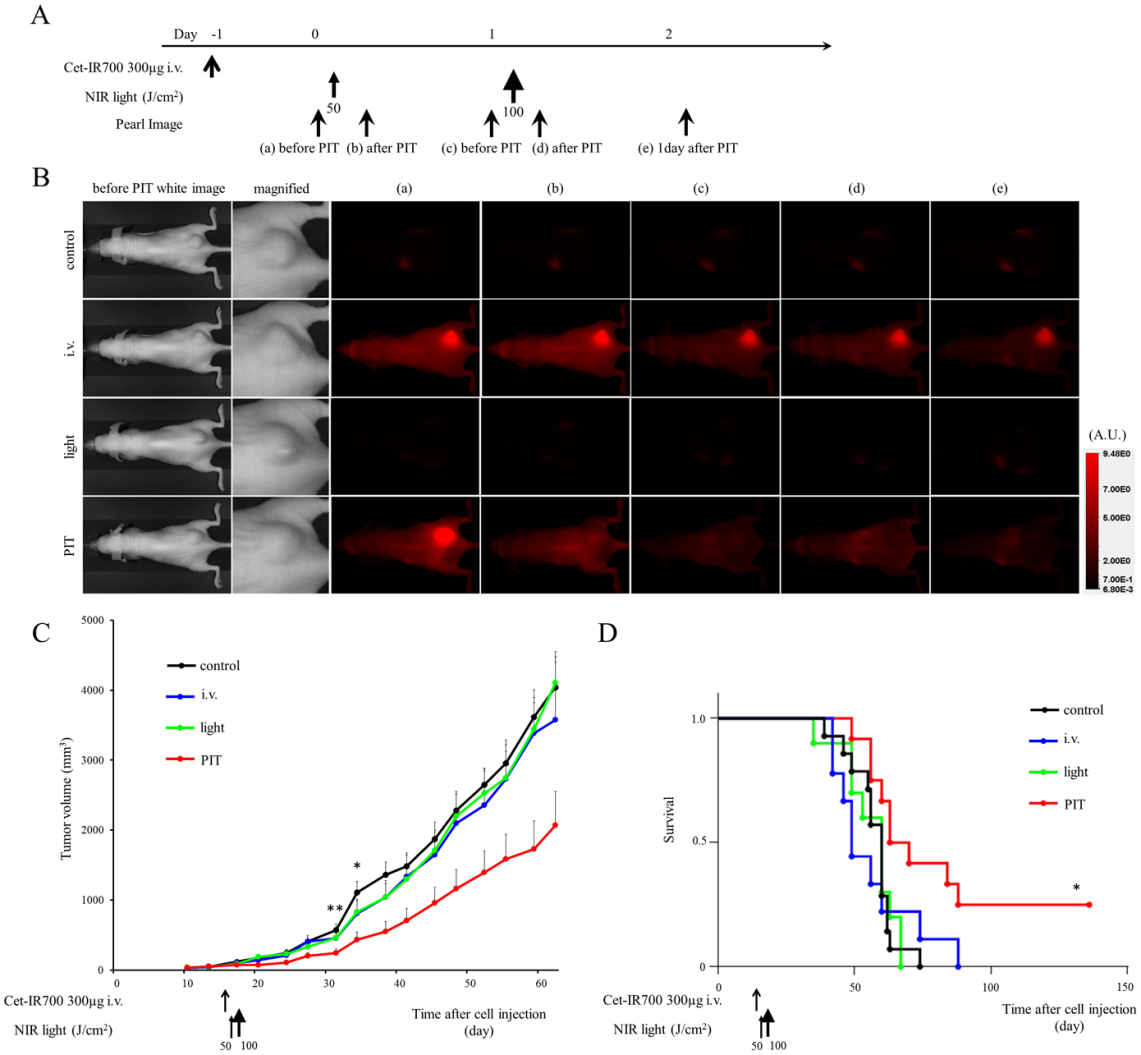
*In vivo* effect of NIR-PIT for MDAMB231 tumor. (A) NIR-PIT regimen. Fluorescence images were obtained at each time point as indicated. (B) In vivo fluorescence real-time imaging of tumor bearing mice in response to NIR-PIT. The tumor treated by NIR-PIT showed decreasing IR700 fluorescence after NIR-PIT. (C) Tumor growth was significantly inhibited in the PIT treatment groups with cet-IR700 (n = 10–13, *p < 0.05 vs i.v. group, **p < 0.01 vs control and light only group, Bonferroni’s test with ANOVA). (D) Significantly prolonged survival was observed in the NIR-PIT treatment group with cet-IR700 (n = 10–13, *p < 0.05 vs other groups, by Log-rank test).

**Fig 4:**
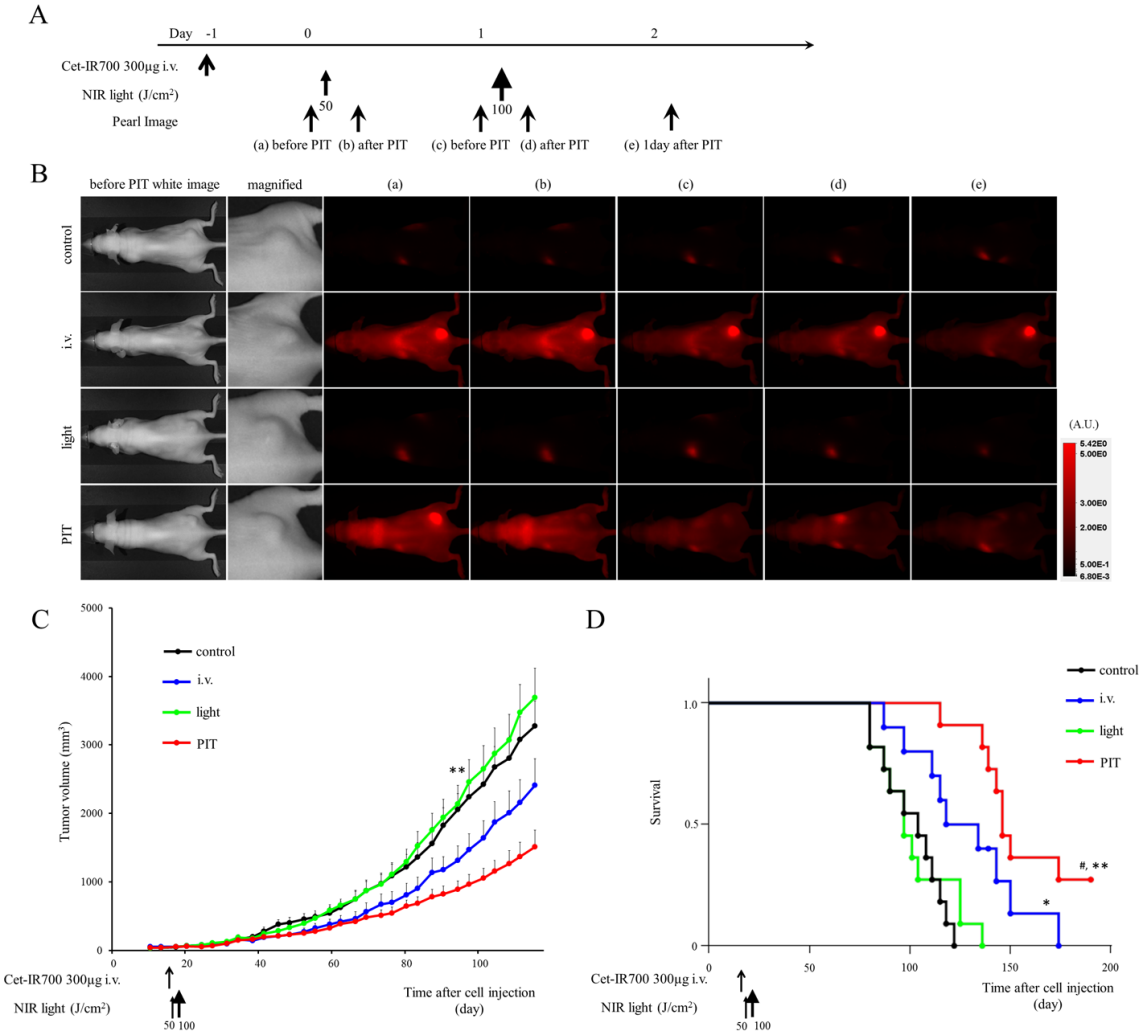
*In vivo* effect of NIR-PIT for MDAMB468 tumor (one shot NIR-PIT). (A) NIR-PIT regimen. Fluorescence images were obtained at each time point as indicated. (B) In vivo fluorescence real-time imaging of tumor bearing mice in response to NIR-PIT. The tumor treated by NIR-PIT showed decreasing IR700 fluorescence after NIR-PIT. (C) Tumor growth was significantly inhibited in the NIR-PIT treatment groups with cet-IR700 (n = 10–11, **p < 0.01 vs control and light only group, Bonferroni’s test with ANOVA). (D) Significantly prolonged survival was observed in the APC i.v. only group and the NIR-PIT treatment group with cet-IR700 (n = 10–11, *p < 0.05 vs control, ^#^ *p* < 0.05 vs i.v. group, **p < 0.001 vs control and light only group, by Log-rank test).

To examine the therapeutic effect of *in vivo* NIR-PIT on MDAMB231 and MDAMB468 cells, serial fluorescence images of tumor bearing mice (n ≧ 10 mice in each group) were assessed before and after each NIR light exposure (day 1 and day 2). One day after injection of cet-IR700, tumor showed higher fluorescence intensity than the tumor with no APC. In both tumors, after exposure to 50 J/cm^2^ of NIR light, IR700 fluorescence signal was compromised due to washing out from dead cells and partial photo-bleaching, but the cet-IR700 fluorescence was retained even 2 days after injection without NIR light exposure (Figs 3B and 4B).

Tumor growth was reduced and survival of MDAMB231 tumor bearing mice was improved in the NIR-PIT treated group (*p* < 0.05) (Fig 3C and 3D). No significant therapeutic effect was observed in the control groups.

In MDAMB468 (one shot NIR-PIT), tumor growth was reduced in the NIR-PIT treatment groups compared with the no treatment and NIR only groups (*p* < 0.01), but there was no significant difference compared with the APC i.v. only group (Fig 4C). Significantly prolonged survival was observed in NIR-PIT treated groups (*p* < 0.05 vs APC i.v. only group, *p* < 0.001 vs control and NIR only group). Mice injected with cet-IR700 alone also showed prolonged survival (*p* < 0.05). No skin necrosis and toxicity of APC was observed in all groups.

### *In vivo* NIR-PIT: multiple shot regimens for MDAMB468 tumor models

In the one shot NIR-PIT regimen for MDAMB468, 300 μg of cet-IR700 i.v. no NIR light group showed small therapeutic effects. To distinguish the influence of APC by itself and the NIR-PIT therapeutic effect, we investigated multiple shot regimens for MDAMB468 tumor models with the same total dose of APC.

**Fig 5:**
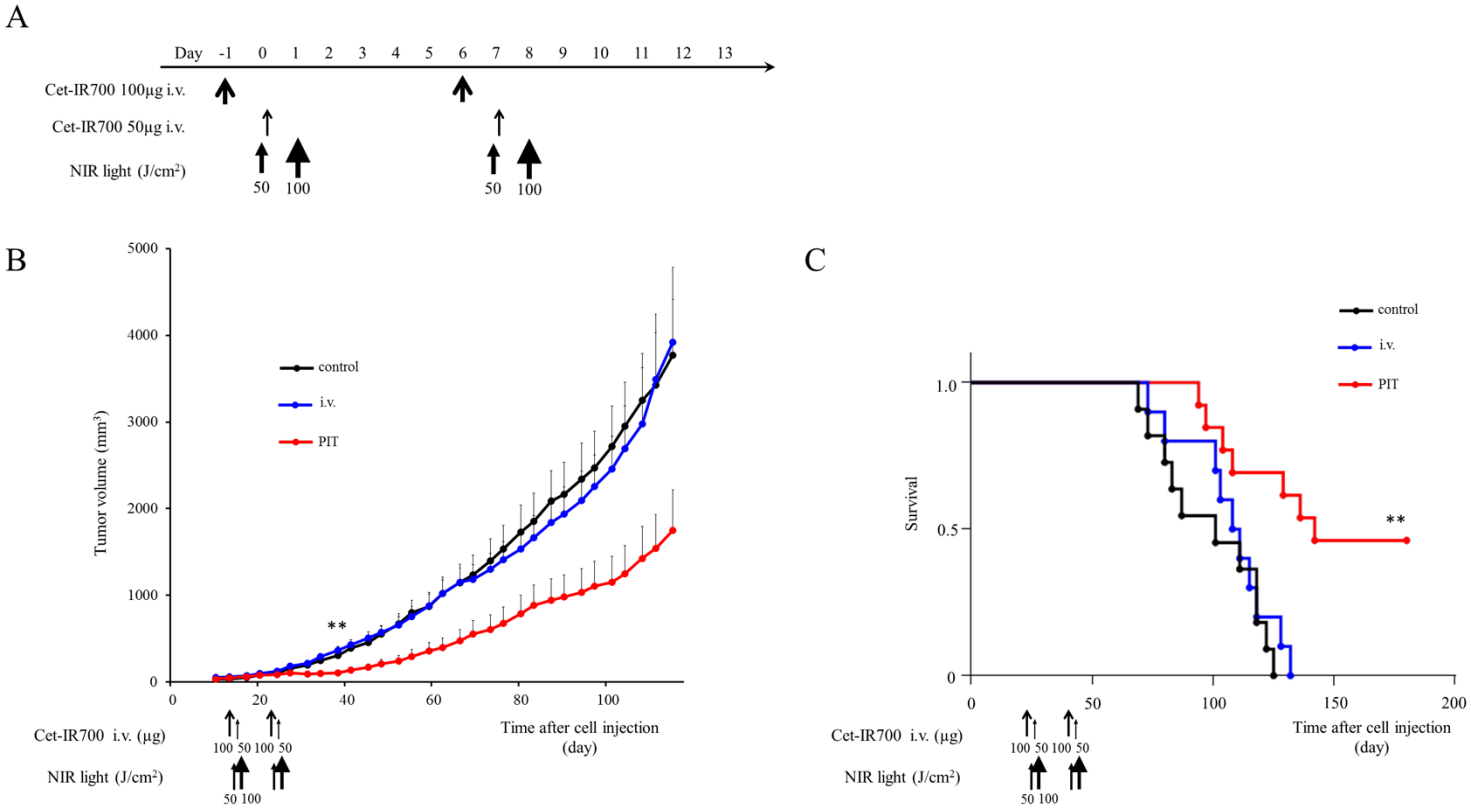
*In vivo* effect of PIT for MDAMB468 tumor (“two split” NIR-PIT). (A) NIR-PIT regimen. (B) Tumor growth was significantly inhibited in the NIR-PIT treatment groups (n = 10–13, **p < 0.01 vs other groups, Bonferroni’s test with ANOVA). (C) Significantly prolonged survival was observed in the APC i.v. only group and the NIR-PIT treatment group (n = 10–13, **p < 0.01 vs other groups, by Log-rank test).

In the “two split” NIR-PIT regimen, tumor growth was significantly inhibited in the NIR-PIT treatment groups compared with the other groups (*p* < 0.01) (Fig 5C), and significantly prolonged survival was achieved in the NIR-PIT group (*p* < 0.01 vs other groups) (Fig 5D). No significant tumor growth reduction or prolonged survival was observed in the control groups including the APC i.v. only or in mice receiving the NIR light only.

**Fig 6:**
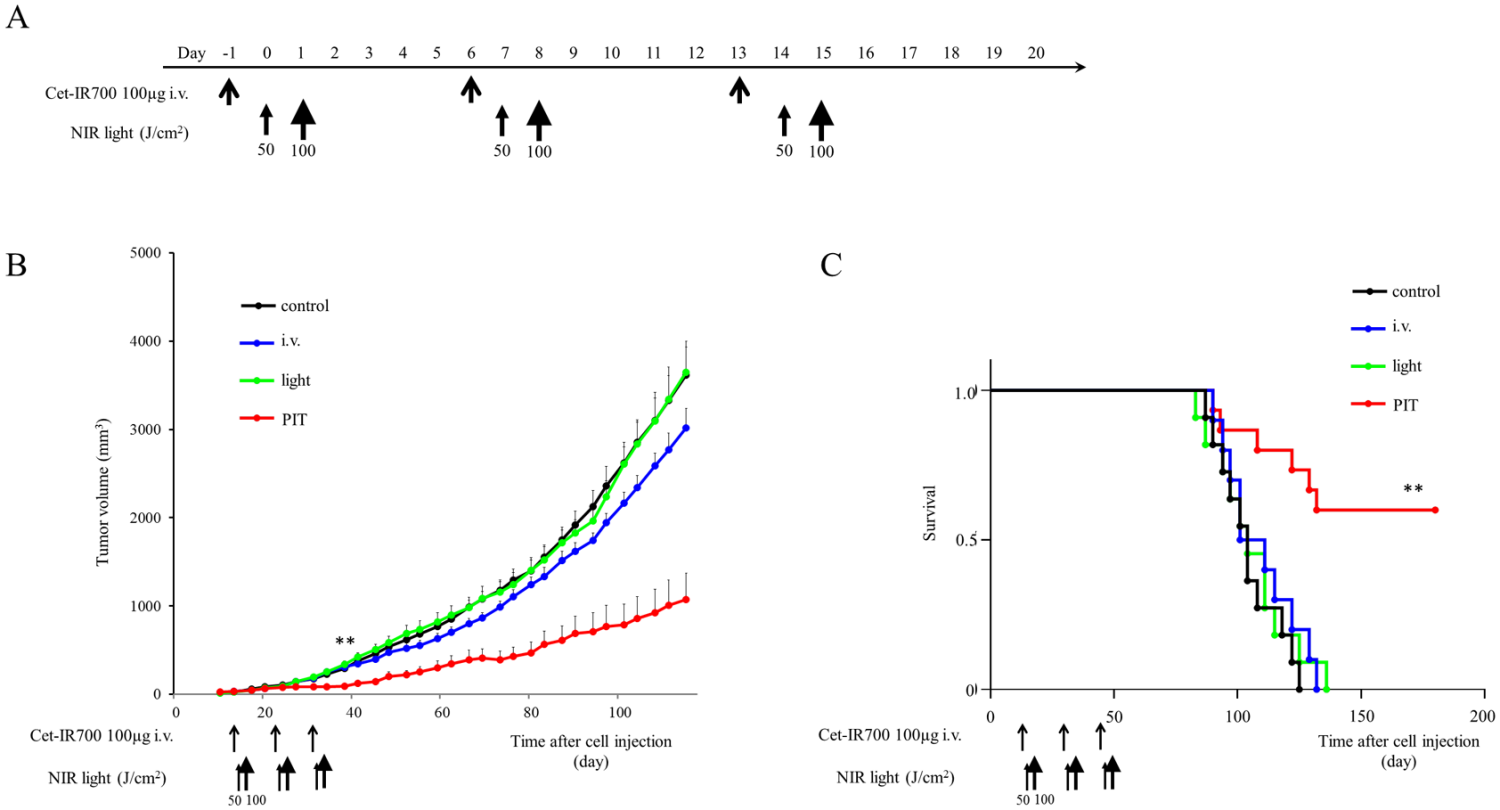
*In vivo* effect of NIR-PIT for MDAMB468 tumor (“three split” NIR-PIT). (A) NIR-PIT regimen. (B) Tumor growth was significantly inhibited in the NIR-PIT treatment groups (n = 10–13, **p < 0.001 vs other groups, Bonferroni’s test with ANOVA). (C) Significantly prolonged survival was observed in the APC i.v. only group and NIR-PIT treatment group (n = 10–13, **p < 0.001 vs other groups, by Log-rank test).

Similarly, tumor volume was reduced and survival was prolonged significantly in the “three split” NIR-PIT regimen (*p* < 0.001 vs other groups) (Fig 6C and 6D). No significant tumor growth reduction or prolonged survival was observed in the control groups including APC i.v. only or in mice receiving NIR light only.

The proportion of mice whose tumors completely disappeared was 9% (1/11) with one shot NIR-PIT, 15% (2/13) with “two split” NIR-PIT, and 38% (5/13) with “three split” NIR-PIT.

These results suggested that split regimens are a good strategy to enhance the NIR-PIT therapeutic effect.

## Discussion

Tumor growth reduction and survival improvements were found in the NIR-PIT treatment groups. These results suggested that NIR-PIT was effective regardless of expression of EGFR. In all treatment regimens, NIR-PIT reduced the rate of tumor growth, resulting in significantly prolonged survival.

To our knowledge, this is the first demonstration of NIR-PIT therapy in TNBC. The term, “PIT”, was first used in studies employing antibody-hematoporphyrin conjugates over thirty years ago [19]. Antibodies were conjugated to very hydrophobic photosensitizers used for photodynamic therapy (PDT). As a consequence of their hydrophobicity, and the conjugate was poorly targeted and little effect was seen. Although we retain the name photoimmunotherapy, in this case it refers to the conjugation of the photosensitizer, IR700, which is water soluble, and therefore, does not permeate into cells or operate as a photosensitizer by itself. Only when IR700 is conjugated with an antibody and APC binds to target molecules on the cell membrane, can NIR light selectively kill target cells. From the pharmacokinetic point of view, IR700 conjugation minimally alters the pharmacokinetics of the antibody. Therefore, similar to antibodies, these APCs show highly targeted accumulation in the tumor and specific binding to target cells with minimal uptake in normal tissue and minimal binding to non-target cells [11–16]. APCs can be synthesized from virtually any antibody, therefore, NIR-PIT could apply to numerous target molecules across a broad range of tumors. Recently, new type of cancer photo-therapy was reported. Cancer cells expressing specific fluorescent proteins can be treated with exposure of UVC [20, 21]. However, the wavelength of UVC is shorter than that of NIR, therefore, UVC light does not penetrate deep into tissue. Furthermore, fluorescent proteins should be genetically transfected into cancer cells *in vivo*. Therefore, we think NIR-PIT would be technically simple and easy.

TNBC is defined by the lack of expression of three critical receptors ER, PR and HER2 [22,23]. Although 50–70% of tumors of the TNBC subtype of breast cancer express EGFR, the therapeutic efficacy of EGFR-targeting agents has been disappointing and despite cytotoxic chemotherapy, the outcome remains poor [9,10,24,25]. NIR-PIT shows highly target specific cytotoxity, and NIR light can be easily applied to primary breast cancers transcutaneously, therefore NIR-PIT is a promising method of treating EGFR expressing TNBC. Here, we demonstrate tumor growth suppression and survival prolongation in mice bearing both MDAMB231 and MDAMB468 tumors. The one shot regimen of NIR-PIT treatment regardless of expression of EGFR showed benefits. According to our results, in high EGFR expressing MDAMB468 tumors, the group of 300 μg of cet-IR700 i.v. only exhibited a small but significant treatment effect compared with other control groups probably because 300 μg of cet-IR700 is a commonly used, single therapeutic antibody dose of cetuximab. After only one shot of NIR-PIT an additional effect was seen. However, to minimize the influence of the APC and maximize the NIR-PIT therapeutic effect, we decided to perform further studies with MDAMB468 tumor models using multiple shots.

In previous work, EGFR-targeted NIR-PIT with a repeated regimen was able to cure >80% of highly expressing A431 tumors [12], so we investigated a split NIR-PIT regimen (total cet-IR700 dose is the same as the one shot NIR-PIT) for MDAMB468 tumor. There are no statistical differences among one shot NIR-PIT and two or three split NIR-PIT on eitther tumor growth or survival curves. However, the NIR-PIT therapeutic effect was improved by repeated APC dosing and light exposures. Side effects were not observed in mice after NIR-PIT partly because cetuximab did not cross react with any murine proteins. However, one shot 300 μg cetuximab is relevant to a therapeutic, single dose for mice, therefore, we observed a small but significant therapeutic effect with APC i.v. alone. When applied to human patients, smaller doses of APC will reduce any side effect.

As additional effect of light therapy, it has been reported that conventional PDT can enhance the delivery of various nanosized or macromolecular drugs up to 3-fold compared with control tumor after PDT when applying low light dose [26–28]. These reports suggested that PDT causes morphologic changes on endothelial cells or the structure of tumor vasculature, leading to tumor enhanced permeability and retention (EPR). In contrast, during NIR-PIT, the APC is maximally bound to cells in the immediate perivascular space and the rapid cell killing leads to an immediate increase in vascular permeability, allowing the rapid leakage of nanosized particles into the tumor space at up to a 24-fold increase compared to baseline. This has been termed super enhanced permeability and retention or SUPR [29–31]. SUPR effects enable the homogeneous redistribution of subsequent antibody-IR700 conjugates within the tumor after the initial NIR-PIT [30]. Based on SUPR effects, we employed two exposures of NIR light one and two days after APC injection in order to take advantage of SUPR on the second injection.

There are several limitations to this study. One limitation is that it does not compare panitumumab- pan-IR700, with cet-IR700 which shows faster clearance from the circulation and higher hepatic accumulation [32]. The difference between panitumumab and cetuximab might derive from protein class characteristics of cetuximab (13% mouse and 87% human) and panitumumab (100% human) or the difference in species. The pharmacokinetics of cet-IR700 may lead to lower therapeutic effects on the second exposure of NIR light in these NIR-PIT regimens based on SUPR effects. Another caveat in this study is that subcutaneously-growing human tumors in immunodeficient mice do not sufficiently represent clinical cancer. To clarify the pre-clinical effect, superior tumor models such as surgically orthotopic implantation model should be used in the future study [33].

## Conclusions

NIR-PIT is effective against EGFR-positive TNBC in a mouse model and results in slowing of tumor growth, prolonged survival. NIR-PIT may be improved by repeated APC dosing and light exposures.

## Supporting Information

S1 Video NIR-PIT effect for MDAMB231 cells. At first PIT (2 J/cm^2^) was performed. Cellular swelling and rupture of cells were observed in some cells of 231. About 2 minutes later, some 231 cells were dead.
(AVI)

S2 Video NIR-PIT effect for MDAMB468 cells. Immediately after exposure to excitation light (2 J/cm^2^) cellular swelling, bleb formation, and rupture of vesicles representing necrotic cell death were observed. About 2 minutes later, almost all the cells were dead.
(AVI)
